# The PPAR *Ω* Pocket: Renewed Opportunities for Drug Development

**DOI:** 10.1155/2020/9657380

**Published:** 2020-07-01

**Authors:** Åsmund Kaupang, Trond Vidar Hansen

**Affiliations:** Section for Pharmaceutical Chemistry, Department of Pharmacy, University of Oslo, 0316 Oslo, Norway

## Abstract

The past decade of PPAR*γ* research has dramatically improved our understanding of the structural and mechanistic bases for the diverging physiological effects of different classes of PPAR*γ* ligands. The discoveries that lie at the heart of these developments have enabled the design of a new class of PPAR*γ* ligands, capable of isolating central therapeutic effects of PPAR*γ* modulation, while displaying markedly lower toxicities than previous generations of PPAR*γ* ligands. This review examines the emerging framework around the design of these ligands and seeks to unite its principles with the development of new classes of ligands for PPAR*α* and PPAR*β/δ*. The focus is on the relationships between the binding modes of ligands, their influence on PPAR posttranslational modifications, and gene expression patterns. Specifically, we encourage the design and study of ligands that primarily bind to the *Ω* pockets of PPAR*α* and PPAR*β/δ*. In support of this development, we highlight already reported ligands that if studied in the context of this new framework may further our understanding of the gene programs regulated by PPAR*α* and PPAR*β/δ*. Moreover, recently developed pharmacological tools that can be utilized in the search for ligands with new binding modes are also presented.

## 1. Introduction

**Figure 1:**
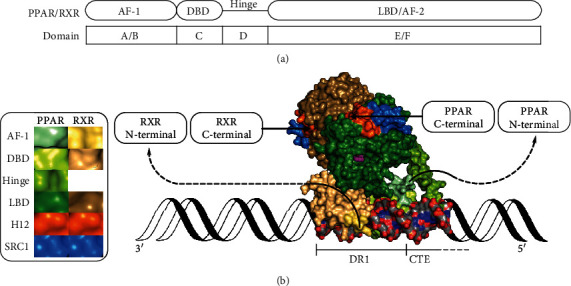
Structural overview of the PPAR/RXR heterodimer. AF-1: ligand-independent activation function 1; DBD: DNA-binding domain; LBD: ligand-binding domain; AF-2: activation function 2; H12: helix 12; SRC1: steroid receptor coactivator 1; DR1: direct repeat 1; CTE: carboxy-terminal extension. (a) Schematic overview of the domains in PPAR/RXR. (b) A molecular surface representation of the structure of the PPAR*γ*:RXR*α* heterodimer bound to rosiglitazone (magenta spheres), 9-*cis*-retinoic acid (not visible), two peptides derived from SRC1, and a DNA fragment. The DNA fragment is shown as a molecular surface (C, grey; O, red; N, blue; P, orange), extended with a cartoon representation (black). The structural data was taken from PDB ID: 3DZY [12] and presented with PyMOL (ver. 1.8.4.0) [13, 14].

The peroxisome proliferator-activated receptors (PPARs) are members of a class of transcription factors whose regulation of gene transcription is modulated by ligand binding—a class also known as nuclear receptors. The three PPAR subtypes described thus far, PPAR*α*, PPAR*β/δ*, and PPAR*γ* (NR1C1, NR1C2, and NR1C3, respectively) [1], are multidomain proteins that each consist of a highly mobile N-terminal domain (domains A/B), a DNA-binding domain (DBD, domain C), a hinge region (domain D), and a C-terminal ligand-binding domain (LBD, domains E/F). Of these, the N-terminal and the C-terminal domains, respectively, contain the ligand-independent activation function 1 (AF-1) and ligand-dependent activation function 2 (AF-2) (Figure 1(a)) [2, 3]. The PPARs are primarily described as acting through heterodimeric complexes with the retinoic X receptors (RXRs) [4]. Upon binding to DNA, each DBD of the PPAR:RXR heterodimer typically interacts with its own half-site of a peroxisome proliferator response element (PPRE) in the promoter or enhancer region of a target gene, e.g., a repeated consensus sequence separated by a single nucleotide—a direct repeat 1 (DR1) element (Figure 1(b)) [5–7]. The PPAR:RXR heterodimer is characterized as *permissive*, in the sense that the binding of ligands in the ligand-binding pocket (LBP) of either receptor can activate transcription. Thus, while the binding of 9-*cis*-retinoic acid (Figure S1) or other RXR agonists to the RXR LBP can positively regulate target genes, the binding of an agonist to the PPAR LBP appears to exert a stronger and dominant role in the activation of the PPAR:RXR heterodimer. Coherently, the binding of agonists to both receptors can synergistically activate transcription [8–11].

In the canonical mechanism, the introduction of an agonist in the PPAR LBP leads to the release of a corepressor protein complex bound to the apo-PPAR:RXR heterodimer through platform proteins such as nuclear receptor corepressor (NCoR), silencing mediator for retinoid and thyroid hormone receptors (SMRT) or SMRT and histone deacetylase-associated protein (SHARP), that either contain or interact with histone deacetylases (HDACs) [15–18]. The holo-PPAR:RXR complex subsequently recruits coactivator proteins such as CREB-binding protein (CBP), steroid receptor coactivator 1–3 (SRC1–3), mediator complex subunit 1 (MED1, TRAP220, or DRIP205), or PPAR*γ* coactivator 1*α* (PGC-1*α*), which in turn recruit other nuclear proteins leading to a coactivator complex that usually displays histone acetylase (HAT) activity. The switch from the action of HDACs to that of HATs increases histone acetylation and leads to the remodelling of chromatin required for the assembly of the functional, multiprotein transcription complex [19–22]. Subsequent transcription of PPAR target genes completes the process known as transactivation. Interestingly, the binding of a PPAR agonist can also lead to the recruitment of certain corepressor proteins, such as receptor-interacting protein 140 (RIP140) [23, 24] or TNFAIP3-interacting protein 1 (TNIP1) [25], that contain receptor-interacting domains (RIDs) similar to those found in coactivator proteins [26].

In contrast to the PPRE-mediated regulation of target gene expression by the PPAR:RXR heterodimer, a holo-PPAR monomer can also interact directly with other transcription factors and attenuate the expression of their target genes—a mechanism called transrepression [27–33]. This fundamentally different mechanism of transcriptional regulation has been shown to be involved in, e.g., the anti-inflammatory effects of PPAR activation [31, 32, 34–36].

The proteins produced upon the expression of PPAR target genes hold key roles in the regulation of lipid and glucose metabolism [37–39]. Consequently, the PPARs have attracted significant attention as possible points of pharmacological intervention in human metabolic diseases. Through the last decades, several members of classical agonist families such as the fibrates (PPAR*α*), the glitazones (PPAR*γ*), and the glitazars (PPAR*α/γ*) have been approved for the treatment of metabolic diseases in humans. However, many of these drugs have since been withdrawn from the market due to the serious side effects that accompanied their clinical use (e.g., carcinogenesis in various tissues, myocardial infarction, loss of bone density, and weight gain) [40–51]. Albeit efficacious at improving selected metabolic parameters in patients, the relative failure of PPAR classical agonists to represent safe treatment options may have caused researchers both in academia and in the industry to abandon further ligand development targeting the PPARs.

Nevertheless, results from the last decade, particularly from the study of the effects of partial and nonagonistic ligands on PPAR*γ*, suggest that the side effects caused by classical agonists and some of the desired, beneficial effects of PPAR*γ* ligation are of separate mechanistic origins. Together, these results form a basis for a new ligand design paradigm, a key concept of which relates to how ligand binding can modulate the occurrence of posttranslational modifications (PTMs) of PPAR*γ*, which in turn lead to particular patterns of gene expression. This and related concepts from the history of PPAR*γ* ligand development are reviewed initially. We then venture to apply these concepts to historic and future ligand development in PPAR*α* and PPAR*β/δ* by highlighting results and ligands that merit renewed attention and further study as tools that can potentially reveal hereto unknown transcriptional profiles of therapeutic relevance.

## 2. PPAR Structure

In addition to the large body of atomic resolution data existing in the public domain on the structures of both apo- and holo forms of the LBDs of the PPARs (domains E/F), our understanding of the structures of the C and D domains, as well as the quaternary organization of domains C–F, is improving [7, 12, 52, 53]. Less is however known about the structural dispositions of the highly mobile N-terminal A/B domains and the AF-1 [54]. Nonetheless, the ability of the AF-1 to induce transcription independently of the AF-2 and of ligand binding has been demonstrated [55–57]. The sequences of the A/B domains also vary significantly between the PPAR subtypes and between the observed splice variants (isoforms) of each subtype [58–61]. Coherently, the AF-1 region has been demonstrated to influence the selectivity with which each PPAR subtype regulates the expression of its target genes [3], but also the degree of transcriptional activation induced by ligand binding [62].

**Figure 2:**
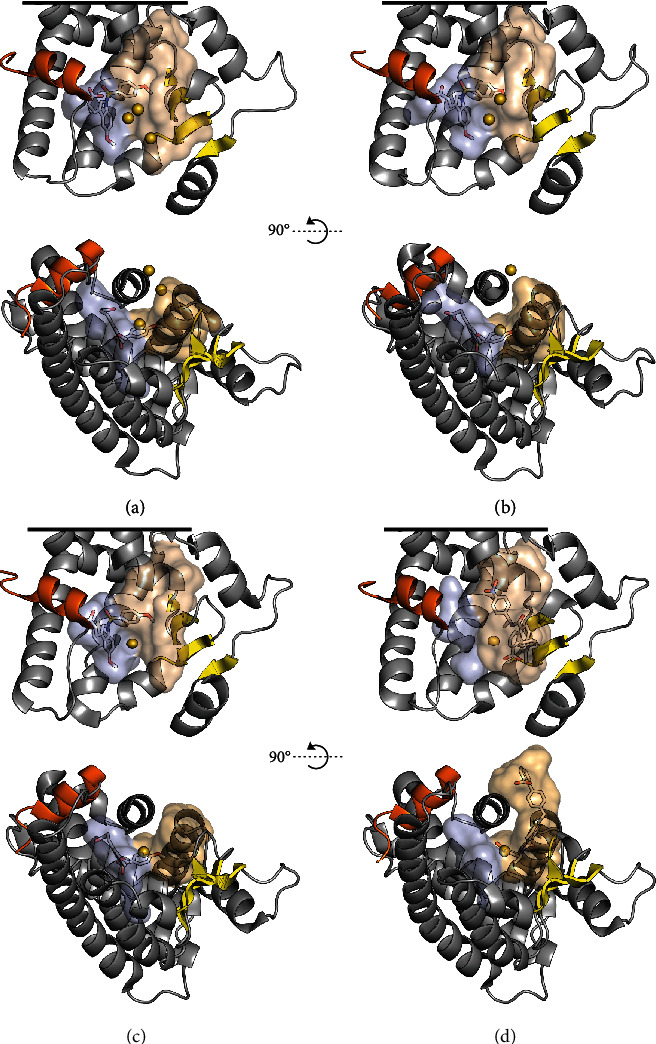
An overview of the shapes of the PPAR*α* (a), PPAR*β/δ* (b), and PPAR*γ* (c, d) LBPs and the subcavities referred to as the AF-2 pocket (light blue) and the *Ω* pocket (beige), lined by helix 12 (orange) and *β*1–*β*4 (yellow), respectively. For clarity, the N-terminal half of H3 and the *Ω* loop are hidden in the front views (top). Also, the visualizations of the LBDs have been truncated (black lines) in order to maximize the visibility of the LBP. Similarly, H2′, the *Ω* loop, and the N-terminal half of H3 are hidden in the top view (bottom). The sulfur atoms of the centrally located cysteines are shown as gold spheres, at 50% of their van der Waals radii. (a–c) PPAR*α*, PPAR*β/δ*, and PPAR*γ* in their respective complexes with indeglitazar (Figure S1) predominantly bound to the AF-2 pocket (PDB ID: 3ET1, 3ET2, and 3ET3, respectively) [100]. (d) PPAR*γ* in complex with SR1664 (Figure S1) bound to the *Ω* pocket (PDB ID: 5DWL) [101]. The LBP surfaces were mapped with a 1.4 Å probe using HOLLOW [102], and the resulting population of probes was truncated at the solvent interface of the *Ω* pocket. The structures and surfaces were visualized in PyMOL (ver. 1.8.4.0) [13, 14].

The structure of the PPAR LBD comprises a sandwich of helices 1–12 (H1–H12), 3-4 *β* strands (*β*1–*β*4), and several prominent loops, with the overall fold being similar to that of other nuclear receptors (NRs) in the steroid hormone receptor superfamily. For the standard PPAR helix numbering scheme, see Uppenberg et al. [63]. The main cavity of the LBD is larger than in other NRs (~1300 Å^3^ [64, 65]) and wraps around the central H3. On either side of H3, the cavity is capped either by the *Ω* loop (the H2′-H3 loop) or by the H12 and the H11–H12 loop. The subcavities on either side of H3 extend along its axis and are additionally limited by the H1-H2 loop, the *β* sheet region, H2′, H5, and H6, on the one side, and by H5, H7, and H11, on the other side. Taking cues from the nomenclature employed by Waku et al. [66], these two subcavities of the PPAR LBP will be referred to as the *Ω* pocket and the AF-2 pocket, respectively (Figure 2). Comparing with more recent literature on PPAR*γ*, these cavities roughly align with the regions referred to as the alternate- or allosteric binding site and the orthosteric binding site, respectively [67, 68]. Overall, the LBPs of the PPARs can be regarded as T- or Y-shaped and they have consequently been divided into three arms [69]; arm I reaches into the AF-2 pocket, while arms II and III largely constitute the *Ω* pocket. The residue numbers given in this text refer to the UNIPROT canonical isoforms for each of the known human PPARs: hPPAR*α*1 (Q07869-1), hPPAR*β/δ*1 (Q03181-1), and hPPAR*γ*2 (P37231-1) [1]. For comparison with earlier literature using, e.g., hPPAR*γ*1 numbering, these residue numbers are given in parentheses were applicable.

On H3, at the interface between the *Ω* pocket and the AF-2 pocket, the PPARs host a region of conserved cysteine residues. The cysteine that is conserved across all three PPARs (hPPAR*α*: Cys276, hPPAR*β/δ*: Cys249, and hPPAR*γ*2(*γ*1): Cys313(285)) is located behind H3 and points into the narrow neck between the *Ω* pocket and the AF-2 pocket (Figure 2) [1, 70]. While this cysteine is demonstrably nucleophilic in PPAR*β/δ* [71–75] and PPAR*γ* [66, 67, 71, 76–84], the eventual nucleophilicity of the corresponding Cys276 in PPAR*α* appears to be surpassed by its neighbour Cys275 [76], which is located on the side of H3 that faces the *Ω* pocket. On the solvent-exposed side of H3, PPAR*α* and PPAR*β/δ* contain additional cysteines (Cys278 and Cys251, respectively), the reactivities of which have not been established (Figure 2).

## 3. Classical PPAR Agonism, Antagonism, and beyond

Structurally, the dissociation of a corepressor protein complex and association with a coactivator protein complex appears to be related to the formation of a tighter groove between H3, H4, and H12, suited for the binding of the RIDs of a given coactivator protein, but that is unable to accommodate the slightly longer RIDs of typical corepressor proteins [64, 85–88]. As the outer surface of H12 is central to the binding of coactivator proteins, many of the known PPAR ligands have either been observed to, or have indeed been designed to, stabilize H12 through interactions with a conserved hydrogen bonding network in the AF-2 pocket, involving conserved tyrosine and histidine residues on H5, H11, and H12 [89].

Access to the AF-2 pocket through the binding of the head groups of classical agonistic ligands has also opened for the development of ligands that display H12-mediated antagonism [90]. These ligands destabilize H12 through perturbation of the AF-2 pocket hydrogen bonding network or by introducing sterically demanding moieties in the AF-2 pocket [86, 90–96]. In PPAR*γ*, interactions with other nearby residues, such as Phe310(282) and Phe391(363), have also been implicated in the mode of action of this class of antagonistic ligands [97, 98]. Based on the observed conformations and folding states of H12 in the few available X-ray crystallographic structures of complexes with such ligands, they appear to disrupt the stable docking of H12 onto the core of the LBD and thus the formation or stabilization of the coactivator-binding groove [85, 88, 99]. In the Phe310/Phe391-interacting class of PPAR*γ* antagonistic ligands, more subtle interactions or alternative binding modes may be involved, which in turn affect the conformational populations of H12 [97, 98].

Some of the reported antagonistic ligands display inverse agonism in that they cause the PPAR-mediated transcription to fall below basal levels in a given model system or assay [91, 94, 95, 97, 103]. In similarity to other reported PPAR inverse agonists [104–106], the observed subbasal transcription levels are likely reflected in the tendency of such ligands to strengthen the interactions of the PPARs with corepressor proteins, such as NCoR and SMRT, compared to those of the apo-PPARs [85, 88, 93, 103–106].

Considering LBD conformational dynamics, ligand binding leads to changes in the conformational populations of the LBD, as observed by nuclear magnetic resonance (NMR) spectroscopy [68, 86, 87, 97, 107–111], hydrogen-deuterium exchange coupled to mass spectrometry (HDX-MS) [68, 97, 101, 107, 112–117], and molecular dynamics (MD) simulations [86, 87, 96, 118, 119]. In PPAR*γ*, analyses of such structural ensembles have demonstrated that the large conformational diversity observed in apo-PPAR*γ*, in particular that of H12, is strongly reduced upon interaction with classical agonists. In contrast, upon treatment with less potent agonists, partial- and nonagonists, H12 still populates several minima [87, 107]. Coherently, high H-D exchange rates have been observed for H12 in PPAR*γ* treated with an inverse agonist, compared to those of H12 in apo-PPAR*γ* [97]. Notably, the motions of the *Ω* loop in holo-PPAR complexes have also been suggested to affect the conformational populations of H12 [78, 118, 119].

During the last decade, evidence has accumulated on the toxicity of clinically employed PPAR*α* and PPAR*γ* classical agonists, such as certain fibrates [40, 41], glitazones [42–46], and glitazars [47–51], as well as that of a PPAR*β/δ* classical agonist in rodents [120, 121]. Combined with the knowledge of their common capacity for stabilization of H12, the frequently observed undesirable effects of these ligands could be interpreted as signs of a mechanism-based toxicity. Furthermore, as findings from the study of PPAR*γ* demonstrate that classical agonism is not required to attain therapeutically relevant transcriptional outcomes (see Section 5), recent ligand development targeting PPAR*γ* has aimed at avoiding ligands that strongly stabilize H12 [97, 122–127]. And while such nonclassical ligands for PPAR*γ* are becoming numerous (Section 5.3), there is a scarcity of similar ligands for PPAR*α* and PPAR*β/δ* (see Sections 6.1 and 7.1).

To amend this, the accumulated experience with the functional effects of ligand interaction with H12, delineated above, may serve as a guide for the future design of partial- and nonagonistic PPAR ligands. Ideally, these ligands should not cause a supraphysiological stabilization of H12, but may rather seek to achieve therapeutically relevant effects, e.g., by influencing PPAR posttranslational modifications (PTMs) (Section 4).

## 4. PPAR Posttranslational Modifications

Ligand binding is far from the only event that affects the activity and physiological roles of the PPARs during their lifetime. The PPARs are observed to be subject to a number of covalent modifications that are common to the nuclear environment and that, as such, are also found on other transcription factors and on histone proteins [128, 129]. So far, the PTMs observed in the PPARs include O-phosphorylation, O-GlcNAcylation, *N*-acetylation, *N*-SUMOylation, and *N*-ubiquitination [130–133]. The investigation of how each of these PTMs modulates protein function is among other things complicated by the effect one PTM can have on another. This encompasses the possibility of a direct competition between PTMs like O-phosphorylation and O-GlcNAcylation of the same serine, threonine, or tyrosine residue [134], as well as between PTMs such as *N*-acetylation, *N*-SUMOylation, and *N*-ubiquitination, which may compete for lysine residues [135–139]. PTMs can also operate in positive- or negative synergy, as in the case of phosphorylation-dependent SUMOylations [140–142] or as observed in the crosstalk between PTMs occurring on lysine or arginine residues, and the phosphorylation of nearby serine or threonine residues [143].

The PTMs that occur in the N-terminal domains of the PPARs can affect both their ligand-independent and their ligand-dependent regulation of gene expression [130–133]. However, while data exist on the influence of some of these PPAR PTMs on the degree of transactivation induced by ligand binding, less is known about the degree to which ligand binding affects the propensity of each of the PPARs towards undergoing such PTMs. From a medicinal chemistry perspective, this question may be of particular interest for PTMs occurring in the LBD, as the magnitude of the influence of ligand binding on the conformational populations of the LBD and thus on the propensity of the LBD to undergo a certain PTM could be larger than for distant regions, such as the N-terminal domain. On the other hand, conformational changes in the LBD may also lead to altered interdomain contacts that in turn can mask or unveil distant PTM sites. Thus, a modulation of the transcriptional outcome by PTMs that occur in the LBD may either be mediated by differential coregulator recruitment [116, 144, 145] or by altered interdomain contacts between the PPAR LBD, the PPAR:RXR DBDs [11, 146], or their N-termini. Additionally, such changes in the conformational populations of the PPARs may influence the promoter binding of PPAR:RXR-heterodimers [7, 12, 118] or the transrepressive activity of PPAR monomers [28–30].

**Figure 3:**
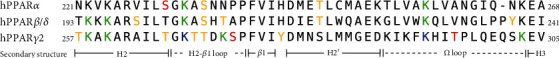
Sequence alignment of the H2-H3 region of the human PPARs, annotated with experimentally observed Ser/Thr/Tyr phosphorylations (red) and Lys acetylations (blue) [152] and with the same PTMs predicted using the tools [147–151] listed in Section 4 (orange and green, respectively). Notably, the employed tools also identified the experimentally observed PTMs.

Most of the knowledge on PPAR PTMs stems from studies of PPAR*γ* and PPAR*α*, while less is known about PTMs occurring in PPAR*β/δ* [130–132]. Interestingly, a large number of consensus PTM sites can be found in the primary sequences of all three PPARs using PTM consensus site mapping algorithms such as GPS-PAIL [147] (*N*-acetylation), PhosphoNET [148], PhosphoMotif Finder [149], or NetPhos [150, 151] (*O*-phosphorylation) (Figure 3). Meanwhile, databases of experimental PTM data, such as PhosphoSitePlus [152], contain records of several PTMs (not limited to phosphorylation), some of which occur in the PPAR LBDs. Among these, some ligand-sensitive PTMs occurring in the PPAR LBDs have been identified and studied experimentally, such as the phosphorylation of PPAR*γ* Ser273, the acetylation of PPAR*γ* Lys268/Lys293, and the phosphorylation of PPAR*α* Ser179/Ser230 (Figure 3 and Sections 5.1, 5.2, and 6, respectively). Guided by data obtained from studies of these PTMs, their therapeutic relevance and the mode of action of the ligands that influence their occurrence are discussed in the following sections.

## 5. New Directions in PPAR*γ* Pharmacology Guided by Relationships between Ligand Binding and PTMs

Among the beneficial effects of PPAR*γ* activation by classical agonists, such as the thiazolidinediones (TZDs) used in the treatment of type II diabetes mellitus (T2DM) and other morbidities associated with obesity, two important axes have been recognized: firstly, improved insulin sensitivity and glucose tolerance, and secondly, increased energy expenditure in white adipose tissue [153, 154]. While historically the structural and mechanistic bases for each of these effects have been unclear, the discoveries and studies of the PTMs introduced above have greatly improved our understanding of their separate origins.

### 5.1. Posttranslational Phosphorylation of Ser273(245) in the PPAR*γ* LBD

In 2010, following the identification of a consensus site for phosphorylation by cyclin-dependent kinase 5 (Cdk5) in PPAR*γ*, Choi et al. demonstrated that Cdk5 indeed phosphorylates Ser273 and that this PTM was associated with insulin resistance in obese mice and humans [116]. The authors could also show that tumor necrosis factor (TNF*α*) induced this phosphorylation, indicating a link between this PTM and the inflammatory state of the obese mice. Using a nonphosphorylatable Ser273Ala mutant, Choi et al. further demonstrated that Ser273 phosphorylation was linked to a reduction in the expression of a subset of PPAR*γ* target genes, including genes linked to insulin sensitivity such as adipsin and adiponectin [116]. The importance of this discovery was amplified by two concurrent findings: firstly, ligand binding decreased phosphorylation at Ser273 and secondly, the ability of a ligand to inhibit Ser273 phosphorylation did not correlate with its ability to induce adipogenesis or transcription in PPAR*γ* reporter gene assays [116, 122]. These results were also in line with observations made previously by other workers, who had developed PPAR*γ* ligands that were poor inducers of transcription of PPAR*γ* reporter constructs, yet displayed potent insulin-sensitizing activity in isolated rodent cells and *in vivo* [155–161].

Choi et al. later demonstrated that a selective interaction of PPAR*γ* phosphorylated at Ser273 (pSer273-PPAR*γ*) with the coregulator protein thyroid hormone receptor-associated protein 3 (Thrap3) underlies the diabetogenic gene dysregulation [162]. In contrast, dephosphorylated PPAR*γ* appears to interact with the coactivator SRC3 [116, 144]. The same group further demonstrated that in the absence of Cdk5, extracellular signal-regulated kinase (ERK) can directly phosphorylate Ser273 in PPAR*γ* and that this phosphorylation is similarly inhibited by PPAR*γ* ligands. In this work, Banks et al. also revealed Thr296 as a novel site of Cdk5 phosphorylation in PPAR*γ* (Figure 3) [163].

Taken together, the above-described findings strongly suggested that an important part of the antidiabetic effects of PPAR*γ* ligands was not linked to their potency as classical agonists but rather to their inhibition of Ser273 phosphorylation [116]. Against the background of the potentially life-threatening side effects observed in the clinical use of PPAR*γ* classical agonists such as the glitazones [42–46], that in comparison strongly stabilize H12 [114, 115], these findings launched a new era for PPAR*γ*-targeting pharmacotherapeutics for the treatment of metabolic disease, in which the focus has shifted towards the development of potent inhibitors of Ser273 phosphorylation, that display little or no classical agonism [123, 127, 164–166].

The discoveries described above were complimented by results from Li et al., who demonstrated that the interaction of Cdk5 with PPAR*γ* was enhanced by interaction of PPAR*γ* with the corepressor protein NCoR [144]. This effect was also observed in adipocyte-specific NCoR knockout mice, in that they displayed decreased levels of pSer273-PPAR*γ* [144]. Together, these findings suggest that an inhibition of PPAR*γ* association with NCoR may be a necessary aspect of the antidiabetic mode of action of PPAR*γ* ligands (see also Section 5.2). Interestingly, Guo et al. recently demonstrated that for optimal repression of PPAR*γ*, but not of PPAR*α* and PPAR*β/δ*, both NCoR and SMRT make use of the corepressor complex component G protein pathway suppressor 2 (GPS-2) [167].

### 5.2. Posttranslational Acetylation of Lysine Residues in the PPAR*γ* LBD

The interaction of PPAR*γ* with NCoR has also been central to the study of another ligand-sensitive PTM, namely, the acetylation of Lys268 (Lys238, mPPAR*γ*1) [145, 168] and Lys293 [145], which are located in the H2-*β*1 loop, close to Ser273, and in the *Ω* loop, respectively (Figure 3). Qiang et al. demonstrated that acetylation of both these lysine residues promoted PPAR*γ* binding to NCoR and that this interaction was further strengthened in the presence of the HAT-containing coactivator protein CBP [145]. Using Lys-Gln mutants to mimic lysine acetylation, the authors also found that the acetylation of Lys293, but that not of Lys268, was linked to Ser273 phosphorylation [145]. These results thus paralleled the previous demonstration by Li et al. of the enhanced interaction of Cdk5 with NCoR-bound PPAR*γ* [144].

Intriguingly, treatment of PPAR*γ* with rosiglitazone, which inhibits Ser273 phosphorylation, also leads to deacetylation of Lys268 and Lys293 (among other lysines [168]) by the nicotinamide adenine dinucleotide- (NAD^+^-) dependent deacetylase Sirtuin 1 (SirT1) [145, 169, 170]. The SirT1-mediated deacetylation of PPAR*γ* was first shown by Han et al., who also demonstrated that PPAR*γ* overexpression or troglitazone (Figure S1) treatment could downregulate SirT1 through the binding of an inhibitory PPAR*γ*/SirT1 complex on the *SIRT1* promoter [171]. Nonetheless, Qiang et al. further demonstrated that the SirT1-mediated deacetylation of Lys293 in particular promoted the interaction of PPAR*γ* with the coregulator PR domain zinc finger protein 16 (Prdm16) [145], which in turn upregulated a thermogenic, brown adipose tissue- (BAT-) related gene program in white adipose tissue (WAT) [145, 172, 173]. In further support of a link between the inhibition of phosphorylation of Ser273 and the deacetylation of Lys293, Wang et al. recently demonstrated that treatment of 3T3-L1-derived adipocytes with the Cdk-inhibitor roscovitine (Figure S1) promoted the dissociation of PPAR*γ* from NCoR, its association with SirT1 and Prdm16, and the subsequent expression of BAT-related genes such as uncoupling protein 1 (*Ucp1*) [174]. Similar results were obtained with a nonphosphorylatable PPAR*γ* Ser273Ala mutant [174]. In contrast, Qiang et al. observed that while both a Ser273Ala mutant and a nonacetylatable Lys268Arg/Lys293Arg double mutant could induce adiponectin in differentiated Swiss 3T3 cells, only the Lys268Arg/Lys293Arg double mutant caused an upregulation of *Ucp1* compared to wild-type PPAR*γ* [145]. Indeed, the interplay between PPAR*γ* acetylation and the phosphorylation status of Ser273 appears to be complex; Mayoral et al. observed that a short-term high-fat diet (HFD) led to increased PPAR*γ* acetylation and Ser273 phosphorylation in adipocyte-specific SirT1 knockout (ATKO) mice. However, although a chronic HFD (15 weeks) led to a further increase in PPAR*γ* acetylation, it was accompanied by a decrease in Ser273 phosphorylation. Consistently, the ATKO mice displayed a concomitant increase in the expression of an insulin-sensitizing gene set and were thus better protected against the negative metabolic effects of the HFD, compared to wild-type mice [175].

As mentioned above, treatment of PPAR*γ* with rosiglitazone, which binds to both the AF-2 pocket and the *Ω* pocket, leads to deacetylation of Lys268 and Lys293 [145, 169, 170]. However, despite the proximity of Lys268 and Lys293 to a ligand binding in the *Ω* pocket, Ohno et al. demonstrated that several partial and nonagonistic PPAR*γ* ligands, which bind to the *Ω* pocket and inhibit Ser273 phosphorylation [116], had practically no effect on the upregulation of the brown adipocyte marker *Ucp1* in mice, compared to rosiglitazone [173]. In contrast, the partial agonist telmisartan (Figure S1) [176, 177], which also inhibits Ser273 phosphorylation [178], has been shown to moderately upregulate *Ucp1* [179, 180]. Additionally, although its binding mode in the PPAR*γ* LBP is not known, the partial agonist natural product formonetin (Figure S1) [181] displayed the same ability [182]. In summary, these results indicate that the structural mechanisms through which PPAR*γ* ligands can influence the acetylation status of Lys268/Lys293 and the upregulation of BAT-related genes in WAT, is in need of further study [183, 184].

### 5.3. Effects of Interaction Patterns and Binding Stoichiometries in the PPAR*γ* LBP on Ser273 Phosphorylation and Transactivation

Given that various classical agonists, partial and nonagonists of PPAR*γ* can all be efficacious as, e.g., insulin sensitizers, the observed toxicity of classical agonists in clinical use has emphasized a need to examine the interactions of each of these ligand classes with the PPAR*γ* LBP, in order to establish which interaction patterns are likely to be conducive to desirable effects, such as the inhibition of Ser273 phosphorylation. In this vein, data from techniques such as X-ray crystallography and HDX-MS have demonstrated that a general theme among PPAR*γ* ligands that are capable of inhibiting Ser273 phosphorylation is their binding to the *Ω* pocket, where they interact with the *β*-sheet region, H2′, the *Ω* loop, or H3 [101, 114, 185]. Through analysis of the binding mode of the 2-aminopyridine tail of rosiglitazone (Figure S1) in the *Ω* pocket, and subsequent ligand design, Bae et al. showed that interaction with a region between H3, residues 312-313 (284-285), and the *β*3-*β*4 loop, residues 368-370 (340-342), was conducive to the inhibition of Ser273 phosphorylation [101]. Notably, this region partially overlaps with the probe clusters P4 and P3, identified in a solvent mapping of the PPAR*γ* LBP performed by Sheu et al., a study which also revealed several other possible sites of ligand interaction in the *Ω* pocket [186]. Consistently, a varied set of ligands of synthetic origin display the general interaction pattern outlined above and inhibit Ser273 phosphorylation, such as MRL24 [116], BVT.13 [116], nTZDpa [116], Mbx-102 [116], GQ-16 [115], F12016 [187], and imatinib [112] (Figure S1). Additionally, a range of natural products of diverse origins [188] bind to the PPAR*γ Ω* pocket, some of which have been demonstrated to inhibit Ser273 phosphorylation, e.g., ionomycin [189], pseudoginsenoside F11 [190], amorfrutin 1 [191], and chelerythrine [192] (Figure S1).

The design of ligands for the PPAR*γ Ω* pocket is complicated among other things by the potential of the PPAR*γ* LBP to harbour more than one ligand simultaneously. Thus, although the binding of a single ligand to the PPAR*γ Ω* pocket was observed crystallographically already in the early days of PPAR research [193], multiple ligands have since been observed to occupy the LBP in complexes with ligand : receptor stoichiometries of 2 : 1 [10, 66, 80, 194–198] and 3 : 1 [66, 199]. Additionally, Shang et al. recently identified electron densities in data from PPAR*γ* cocrystals previously thought to be stoichiometric complexes, corresponding to cocrystallized nonanoic acid ligands [200]. Such passenger fatty acids, likely derived from the bacteria in which the PPAR proteins are expressed, have also been observed in PPAR*β/δ* cocrystals [201, 202].

As metabolic sensors, the PPARs are moderately to strongly activated by medium chain fatty acids (MCFAs) [199, 203], long-chain mono- and polyunsaturated fatty acids (MUFAs/PUFAs) and some of their metabolites [65, 204–209], as well as by oxo- and nitro-fatty acids [76–78, 80–82]. The members of the latter two ligand groups bind covalently to the central cysteine residue, Cys313(285) of the PPAR*γ* LBP. In PPAR*γ* reporter gene assays, the degree of transactivation by both MCFAs and MUFAs peak at certain chain lengths [199, 203], possibly reflecting the ligand : receptor binding geometries and -stoichiometries available to a given fatty acid. Furthermore, the simultaneous binding of 15-oxoeicosatetraenoic acid (15-oxoETE, Figure S2) and the serotonin metabolite 5-methoxyindole acetate (MIA, Figure S2) has been observed in the crystal phase. Notably, the maximum transcriptional activity induced by a combination of 15-oxoETE (10 *μ*M) and MIA (100 *μ*M) in a PPAR*γ* reporter gene assay was roughly twice that induced by rosiglitazone (1 *μ*M), while either 15-oxoETE or MIA alone, at the same concentrations, only induced about half the activity of rosiglitazone [66].

In parallel, while treatment of PPAR*γ* with nonanoic acid or docosahexaenoic acid (DHA) (Figure S1) inhibited Ser273 phosphorylation [199], treatment with a mixture of oleic and palmitic acid (Figure S1) [116], with palmitic acid alone [210] or with eicosapentaenoic acid (EPA, Figure S1) [211], appeared to promote this PTM. Interestingly, in the latter study, DHA induced a higher expression of adiponectin at 100 *μ*M than at 200 *μ*M [211].

Considering ligands of synthetic origin, the ligand BVT.13 [159, 212] (Figure S2) was observed to bind to PPAR*γ* in a 1 : 1 stoichiometry in the crystal phase, primarily interacting with H3 and not with H12 [12, 114, 159]. And while HDX-MS experiments did not indicate that BVT.13 treatment stabilized H12 significantly compared to apo-PPAR*γ* [114], the transcriptional response to BVT.13 was 60-80% of that of rosiglitazone, in PPAR*γ* reporter gene assays [114, 159]. BVT.13 is also an inhibitor of Ser273 phosphorylation [116]. In contrast, 10 *μ*M of the ligand GW0072 (Figure S2), which also appears to bind exclusively to the *Ω* pocket, displayed a maximum transcriptional activation of 20% of that induced by rosiglitazone (1 *μ*M, 100%) in a PPAR*γ* reporter gene assay. While data on the ability of GW0072 to inhibit phosphorylation of Ser273 is not available, GW0072 caused dissociation of NCoR from PPAR*γ* [193]. However, it is noteworthy that GW0072 (10 *μ*M) did not induce the expression of neither adipsin nor fatty acid-binding protein 4 (FABP4/aP2) in 10T1/2 cells after up to 6 days, while rosiglitazone (1 *μ*M) strongly upregulated both after 6 days [193].

Examples of negative cooperativity from the binding of multiple ligands of synthetic origin have also been reported. The ligand T2384 (Figure S2) displayed biphasic response curves in coregulator recruitment assays, with reduced recruitment of the coactivator MED1 (DRIP205) and increased recruitment of the corepressor NCoR at higher concentrations [197]. A similar biphasic coregulator recruitment pattern was also observed for the partial agonist telmisartan [176].

The phenomenon of multiple ligation of the PPAR*γ Ω* pocket at higher ligand concentrations is paralleled by the binding of a single ligand in multiple conformations, as observed in the case of, e.g., SR1664 (Figure S1) [97, 101]. In the crystal phase, SR1664 was first shown to bind in a conformation similar to that of classical agonists, in which its interactions with Phe310(282) appeared to prevent it from functioning as an agonist [97, 98]. Bae et al. later demonstrated that SR1664 also binds to the *Ω* pocket (Figure 2(d)) and that this binding mode is likely of greater importance for its inhibition of Ser273 phosphorylation [101].

Taken together, these results paint a complex picture in which the binding of multiple molecules of the same or of different ligands is possible and may result in either positive or negative cooperativity in terms of transactivation. The collective efforts described above also illustrate that while inhibition of Ser273 phosphorylation likely requires interactions with regions of the PPAR*γ Ω* pocket, a single consensus pharmacophore for the design of nonagonistic inhibitors of Ser273 phosphorylation has yet to be firmly established.

### 5.4. Clinical Applications of pSer273 Inhibitors

Interestingly, metabolic disease was not the only condition that could potentially be remediated by partial or nonagonistic PPAR*γ* ligands. In a microarray analysis of the genes regulated by the murine, nonphosphorylatable Ser273Ala PPAR*γ* mutant, Choi et al. revealed that two genes involved in myelination, neuroblast differentiation-associated protein AHNAK (*Ahnak*/desmoyokin) [213] and myelin proteolipid protein (*Plp1)* [214], were also positively regulated [116]. PPAR*γ* has been found in high concentrations in the cerebrospinal fluid of multiple sclerosis (MS) patients [215] and has been suggested to play a role in neuroprotection and remyelination [216, 217]. Although the cluster containing *Ahnak* and *Plp1* was not as strongly upregulated by PPAR*γ* ligands as by the Ser273Ala mutant [116], these findings suggested that inhibitors of PPAR*γ* Ser273 phosphorylation may also be useful in the treatment of inflammation-related neurodegenerative diseases.

This dual therapeutic potential is exemplified in the account of the PPAR*γ* partial agonist CHS-131 (formerly INT131, T0903131, T131, and AMG131, Figure S2) [156, 218, 219]. While CHS-131 (1–10 mg/day) displayed promising results in clinical trials oriented towards the treatment of metabolic diseases (NCT00952445 [220] and NCT00631007 [221]), it was later repurposed for the treatment of relapsing-remitting MS (RRMS) [222]. In 2016, upon completion of a 6-month phase IIb trial in patients with treatment-naïve RRMS, Coherus BioSciences Inc. reported that CHS131 decreased cumulative contrast-enhancing (CE) and T2 lesions, as well as cortical volume loss in the treatment group (NCT02638038) [223, 224]. In the former, metabolism-oriented trials, dose-dependent, yet less severe side effects were observed [220], comparing CHS131 to the PPAR*γ* classical agonist pioglitazone (Figure S2) on parameters such as hemodilution and edema. No bone demineralization was observed with CHS-131, although the study was not powered to statistically evaluate this effect against that of pioglitazone [221]. In the RRMS trial, no serious side effects were noted at the employed effective dose (3 mg/day) [223, 224]. On the background of the gene expression data for rosiglitazone versus that of the weak partial agonist MRL24, reported by Choi et al. [116], these clinical observations can also be interpreted to provide support for the notion that the observed side effects of PPAR*γ* classical agonists stem from the broader gene set they induce.

In the context of this review, the apparent clinical efficacy and safety of CHS-131 are of particular interest since, as a PPAR*γ* ligand, CHS-131 may be characterized as a partial classical agonist based on its observed activation of transcription in various PPAR*γ* reporter gene assays (15-40% of the effect of rosiglitazone) [218, 219, 156]. This could suggest that more completely nonagonistic ligands may display even better safety profiles. Indeed, two studies report that structural modifications to CHS-131 can produce ligands with substantially lower transactivation capacities, without sacrificing affinity for PPAR*γ* [218, 225].

Finally, as suggested by a recent study, a future clinical application of nontoxic inhibitors of PPAR*γ* Ser273 phosphorylation may also include their use as adjuvants to chemotherapy, in cases in which increased levels of pSer273-PPAR*γ*, resulting from DNA damage by cytotoxic agents, leads to reduced sensitivity to the chemotherapy [226].

## 6. Relationships between PTMs and Ligand Binding in PPAR*α*

The available data on relationships between PTMs in the PPAR*α* C-terminal domains (D–F) and ligand binding are limited, but include two reports describing the protein kinase C- (PKC-) mediated phosphorylation of PPAR*α* and its effects on the propensity for ligand binding to confer either transactivation (PPRE-mediated transcription of target genes) or transrepression [227, 228]. Prompted by an earlier result linking the expression of PPAR*α* to that of PKC in rat liver [229], Blanquart et al. demonstrated that the phosphorylation of PPAR*α* at Ser179 and/or Ser230 by PKC increased both basal and ligand-induced transcription of target genes such as carnitine palmitoyltransferase 1 (*CPT1)* and *PPARA* in a human liver cancer cell line [227]. Conversely, pharmacological inhibition of PKC or the use of a nonphosphorylatable, Ser179Ala/Ser230Ala double mutant, reduced the ability of PPAR*α* classical agonists like WY14643 (pirinixic acid) and GW7647 (Figure S3) to induce the expression of these target genes and increased both the basal and the ligand-induced PPAR*α*-mediated transrepression of the basal expression of fibrinogen beta chain (FGB) [230, 231] in HepG2 cells [227]. Similarly, Paumelle et al. could demonstrate that PKC inhibition or use of the PPAR*α* double mutant attenuated the lipopolysaccharide- (LPS-) induced expression of the proinflammatory nuclear factor NF-kappa-B (NF*κ*B) target gene inducible nitric oxide synthase (iNOS) in murine macrophages. The transrepression of LPS-induced iNOS expression was also observed upon treatment of PPAR*α* with the agonist GW9578 (Figure S3) and the 3-hydroxy-3-methyl-glutaryl-coenzyme A (HMG-CoA) reductase inhibitor simvastatin (Figure S3). The authors further demonstrated that while the transrepression of iNOS expression was dependent on PPAR*α* for both these ligands, pretreatment of murine macrophages or neutrophils with simvastatin also interfered with the ability of LPS-induced, immunoprecipitated PKC*α* to phosphorylate a generic target like histone H1 in an *in vitro* assay [228]. Nonetheless, taken together with the stronger transrepressive effect observed upon upstream inhibition of PPAR*α* phosphorylation using PKC inhibitors, the transrepressive effects of PPAR*α* treatment with WY14643, GW7647, or GW9578 alone, compared to DMSO controls, may indicate that ligand binding itself, at least in part, reduces the propensity for PPAR*α* to be phosphorylated by PKC.

Structurally, PPAR*α* Ser179 is located in the hinge region, close to the PPAR*α* DBDs, while Ser230 is located in the H2-*β*1 loop, in similarity to the Cdk5 phosphorylation target Ser273 in PPAR*γ* (Figure 3). Thus, the results described above could indicate that the H2-*β*1 loop is a region of general interest for ligand-sensitive PTMs in the PPAR LBDs.

Interestingly, in a later, independent study by Roy et al., it was confirmed that simvastatin is also a PPAR*α* ligand (EC_50_ = 4.26 *μ*M) [232]. In this study, the authors also performed molecular docking of the simvastatin *δ*-lactone to the PPAR*α* LBP and found that it bound to the *Ω* pocket, where it interacted with Leu331 and Tyr334 of *β*3. Subsequently, site-directed mutagenesis of these residues (Leu331Met, Tyr334Asp) and evaluation in a PPAR*α* reporter gene assay revealed that simvastatin was unable to activate transcription through the Leu331Met/Tyr334Asp mutant. Notably, similar results were obtained for mevastatin and its 6-hydroxylated, ring-opened analogue pravastatin (Figure S3) [232]. However, the propensity of statin *δ*-lactones to be converted to their 3,5-dihydroxy acids (Figure S3) through hydrolysis or metabolism [233–236] raises the question of whether the observed effects of PPAR*α* treatment with statins stem from the binding of the intact *δ*-lactones, the 3,5-dihydroxy acids, or both.

Nevertheless, Roy et al. demonstrated that treatment of murine astrocytes with simvastatin led to an upregulation of neurotrophin 3 (NT-3) and brain-derived neurotrophic factor (BDNF) which was dependent on cAMP response element-binding protein (CREB), whose expression is in turn regulated by PPAR*α* [232]. The transcriptional regulation of CREB by PPAR*α* appears to play a central role in hippocampal neuron plasticity and spatial memory consolidation in mice [237]. These results suggest a potential for PPAR*α*-targeting ligands that regulate CREB expression in the treatment of neurodegenerative diseases such as Alzheimer's disease [238, 239].

In summary, additional studies are needed to elucidate whether both of the discussed PKC phosphorylation sites in PPAR*α* are involved in the observed effects, to what degree their phosphorylation is sensitive to ligand binding and what the binding modes of such ligands are. Taken together, however, these results illustrate that a refinement of our understanding of the interplay between ligand binding modes, PTMs, and their combined physiological effects may be instrumental towards harnessing the therapeutic potentials of PPAR*α* ligands that do not necessarily share the transcriptional profiles of classical agonists.

### 6.1. PPAR*α* Ligands with Alternative Binding Modes

**Figure 4:**
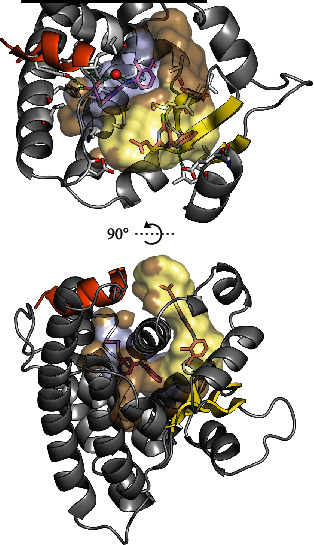
PPAR*α* in complex with two molecules of WY14643 (shown with magenta carbons) taken from PDB ID: 4BCR [119]. H12 is shown in orange, and *β*1-*β*4 are shown in yellow. To illustrate ligand-pocket interactions, the inner surface of the binding pocket is shown in brown with surface areas ≤ 3.7 Å from the ligand binding primarily in the AF-2 pocket highlighted in light blue. Similarly, the contact surfaces of the ligand binding in the *Ω* pocket and under the *Ω* loop are shown in pale yellow. Top: residues ≤ 5.0 Å from the ligands are shown with grey carbons. Plausible hydrogen bonds are indicated with green dashes. The side chain oxygen of Ser280 is shown as a red sphere at 50% of its van der Waals radius. For clarity, the *Ω* loop and the N-terminal half of H3 (residues 252-284) are hidden. Also, the visualization of the LBD has been truncated (black line) in order to maximize the visibility of the LBP. Bottom: a perpendicular view to the LBP illustrating the distance between the WY14643 binding sites. The *Ω* loop is shown in a cartoon representation with two notable helical segments, indicating its stabilization by the binding of the second molecule of WY14643 [119]. The LBP surfaces were mapped with a 1.4 Å probe using HOLLOW [102], and the resulting population of probes was truncated at the solvent interface of the *Ω* pocket. The structures and surfaces were visualized in PyMOL (ver. 1.8.4.0) [13, 14].

So far, few ligands have been reported to display alternative binding modes in the PPAR*α* LBP. In the crystal phase, Bernardes et al. observed that while one molecule of WY14643 bound in a conformation akin to other PPAR*α* classical agonists, a second molecule bound to a novel site under the *Ω* loop (Figure 4). This binding mode strongly stabilized the *Ω* loop, as observed both in the crystal phase and with MD simulations [119]. Of particular interest in the context of the studies on the possible PKC-mediated phosphorylation of PPAR*α* Ser230 described above, MD simulations also demonstrated that the binding of the second molecule of WY14643 stabilized the Ser230-containing H2-*β*1 loop (Figure 3) [119].

Finally, during the development of the *Ω* pocket-binding PPAR*γ* ligand BVT.13 [159, 212], described in Section 5.3, three additional ligands, BVT.762, BVT.763, and Compound 5d (Figure S3), also displayed binding to PPAR*α* (*K*_*i*_ = 25 *μ*M, 20 *μ*M, and 19 *μ*M, respectively) and induced transcription in a PPAR*α* reporter gene assay with EC_50_ = 5 *μ*M, 3.8 *μ*M, and 2.5 *μ*M, respectively [212]. Although no data is available on their binding modes in the PPAR*α* LBP or their influence on PPAR*α* PTMs, the binding of BVT.13 to the PPAR*γ Ω* pocket suggests that these ligand structures may be of interest in the development of ligands for the PPAR*α Ω* pocket.

## 7. PPAR*β/δ* PTMs and Ligands with Nonclassical Binding Modes

To the best of our knowledge, the only records to date of experimentally determined PTM-like modifications of the PPAR*β/δ* LBD are contained within a curated entry in the PhosphoSitePlus database. This entry denotes the immunohistochemical- and mass spectrometrical detection of PPAR*β/δ* apparently phosphorylated at Thr252, Thr253, and Thr256 on H3 by ribosomal protein S6 kinase alpha-3 (RPS6KA3), also known as p90 ribosomal S6 kinase 2 (RSK2) [240]. Consequently, no further details exist on the function or ligand sensitivity of these PTMs. Structurally, the partially buried localization of Thr252, Thr253, and Thr256 raises the question of whether they are indeed plausible PTM sites. It is noteworthy, however, that RSK2 has been reported to interact with an overlapping region in the human estrogen receptor *α* (hER*α*, residues 326-394, including H1, the H1-H3 loop, H3, H4, and H5) [241].

### 7.1. PPAR*β/δ* Ligands with Alternative Binding Modes

**Figure 5:**
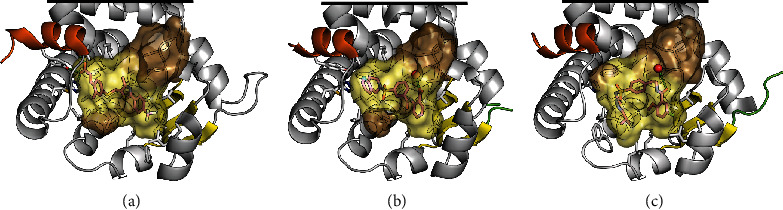
The LBP of PPAR*β/δ* in complex with ligands (shown with magenta carbons). To illustrate ligand-pocket interactions, the inner surface of the binding pocket is shown in brown with surface areas ≤ 3.7 Å from the ligand highlighted in pale yellow. Residues ≤ 5.0 Å from the ligand are shown with grey carbons. Plausible hydrogen bonds are shown with green dashes. H12 is shown in orange, and *β*1-*β*4 are shown in yellow. The unresolved termini of the H2–*β*1 loop are shown in light green. For clarity, the *Ω* loop and the N-terminal half of H3 (residues 224–257) are hidden. Also, the visualizations of the LBD have been truncated (black lines) in order to maximize the visibility of the LBP. (a) Classical agonist, GW0742 (PDB ID: 3TKM) [245]. (b) Partial agonist GW9371 (PDB ID: 3DY6) [242]. The side chain oxygen of Thr252 is shown as a red sphere at 50% of its van der Waals radius. (c) Compound 6 (PDB ID: 2XYX) [243]. The LBP surfaces were mapped with a 1.4 Å probe using HOLLOW [102], and the resulting population of probes was truncated at the solvent interface of the *Ω* pocket. The structures and surfaces were visualized in PyMOL (ver. 1.8.4.0) [13, 14].

In studies of PPAR*β/δ*, ligands that display binding modes other than those of classical agonists have also been described. Shearer et al. reported a series of potently binding partial agonists, some of which were intriguingly poor inducers of transcription. Compound 34, Compound 13, and Compound 14 (Figure S4) displaced a radioligand with IC_50_ = 13 nM, 3 nM, and 10 nM, respectively. In a cell-based reporter gene assay, Compound 34 and Compound 13 displayed an EC_50_ = 0.2 *μ*M and an EC_50_ = 1.3 *μ*M, with 29% and 37% maximal activation, respectively. The transcriptional activation observed with Compound 14, on the other hand, was below the sensitivity threshold of the reporter gene assay (~20%). In comparison, the classical agonist GW501516 (Figure S4) displayed an IC_50_ = 5 nM and an EC_50_ = 3 nM (98% maximal activation) in the same assays. In a crystal structure of PPAR*β/δ* in complex with the original high-throughput screening (HTS) hit, GW9371 (Figure S4, EC_50_ = 1.3 *μ*M, 61% maximal activation), the ligand bound around H3, with its tetrahydroisoquinoline moiety protruding into the AF-2 pocket [242]. In contrast to the polar interactions common to the carboxylic acid head groups of classical agonists such as GW0742 (Figure S1), the distal aryl ring in GW9371 appears to display primarily hydrophobic interactions with the surrounding residues, among them Tyr437 of H12 (Figures 5(a) and 5(b)). Compound 13 and Compound 14 are structural analogues of GW9371 in which the hydrogen in the 5-position of its tetrahydroisoquinoline ring (Figure 5(b), teal sphere) has been substituted with a formic acid or a 2-oxyacetic acid, respectively (see Figure S4). While, as the authors suggest, the introduction of these substituents may have affected the membrane permeability of the resulting ligands [242], it is also possible that the diminished agonistic activities of Compound 13 and Compound 14 compared to GW9371 owe to the bulkiness of their AF-2 pocket-binding moieties, which may cause them to display H12-mediated antagonism (see Section 3).

Keil et al. described a series of ligands that interacted with H12 to a lesser degree than the GW series described above, binding in a U-shaped conformation around Cys249 (e.g., Compound 6, Figure 5(c) and Figure S4). From the extensive structure-activity relationships in all three PPARs established by the authors, a relevant ligand in this context was Compound 11j (Figure S4), which displayed an EC_50_ = 120 nM with 22% activation of a PPAR*β/δ* reporter gene assay (100% was set as the maximum achievable activation by GW501516). Importantly, Compound 11j also displayed a high selectivity for PPAR*β/δ* over the other PPARs [243].

In a genome-wide analyses of the transcriptional regulation by PPAR*β/δ* in myofibroblasts treated with a classical agonist such as GW501516 or L-165,041 (Figure S4), or with *PPARD* siRNA, Adhikary et al. demonstrated distinct modes of transcriptional response among PPAR*β/δ* target genes, including their individual ligand inducibilities [244]. If applied to nonclassical ligands such as Compound 14 (above) or Compound 11j, analyses in the same vein could reveal distinct regulatory patterns of therapeutic relevance. In summary, Compound 14 and Compound 11j represent valuable pharmacological tools to compare the transcriptional effects of weak partial agonists to those of classical agonists that actively stabilize PPAR*β/δ* H12.

## 8. Pharmacological Tools and Recent Advances in the Development of Ligands for the PPAR *Ω* Pockets

### 8.1. PPAR*γ*

It has been observed that while treatment of PPAR*γ* with ligands that bind covalently to the central cysteine residue, Cys313(285), such as GW9662 [71] or T0070709 [83] (Figure S2), limits the access to the AF-2 pocket (i.e., antagonizes the action of classical agonists), it does not inhibit the subsequent binding of an additional ligand in the *Ω* pocket [68, 101]. This phenomenon aligns with the numerous previous observations of the capacity of the PPAR*γ* LBP to house multiple ligands simultaneously, as discussed in Section 5.3. Consequently, pharmacological and physiological (re)interpretations of the effects of treatment of PPAR*γ* with this class of antagonists should reflect the possibility of interference from ligands that bind to the *Ω* pocket of the covalently modified receptor [68]. The notion that such interference could be pharmacologically relevant is substantiated by the observation that, in similarity to the synergistic activation observed upon simultaneous binding of multiple MCFAs [199, 203], the binding of a second ligand to PPAR*γ* treated with GW9662 produced a stronger response in a PPAR*γ* reporter gene assay than that observed with the ligand alone [246]. The classical PPAR*γ* agonist MRL20 (Figure S2) has also been demonstrated to retain its ability to activate PPAR*γ* after treatment with GW9662 or T0070907 by binding to the *Ω* pocket in a pose that was markedly different [68] from its crystallographically observed pose in untreated PPAR*γ* (PDB ID: 2Q59) [114]. Thus, to address the “single-sided” antagonism of GW9662 and T0070907, Brust et al. recently designed analogues of these ligands, with bulkier groups protruding into the *Ω* pocket, blocking the binding of MRL20 [67].

On the other hand, the persistent ligand binding ability of the *Ω* pocket in PPAR*γ* treated with GW9662 or T0070907 also opened up the possibility to use the covalently modified protein as a model in which to screen for new *Ω* pocket binders. Indeed, Ohtera et al. developed a method to screen a natural product extract library for the ability of the tested fractions to cooperatively activate transcription in a PPAR*γ* reporter gene assay, upon cotreatment with GW9662 [246]. This allowed the authors to identify a methoxyphenylcinnamic ester ligand and further combine its structure with that of GW9662 to produce a hybrid partial agonist, Compound 5 (Figure S2), that bound covalently to Cys313(285) and likely occupied the *Ω* pocket (supported by molecular modelling) [246]. The ability of Compound 5 to inhibit PPAR*γ* Ser273 phosphorylation was not investigated. In a later study, Bae et al. prepared a series of analogues of GW9662 which were shown by X-ray crystallography to bind covalently to Cys313(285), as well as to occupy the *Ω* pocket [101]. These ligands were designed to place an aryl moiety in a specific region between H3, *β*3, and *β*4 (see Section 5.3). The most promising of the resulting ligands, SB1405 and SB1453 (Figure S2), were shown to be potent inhibitors of Ser273 phosphorylation and practically devoid of classical agonism [101]. These results suggest that the covalent PPAR*γ* partial agonist, L-764406 (Figure S2), reported by Elbrecht et al. in 1999 [84], may also be capable of inhibiting Ser273 phosphorylation. During their studies, Bae et al. also prepared the *N*-methyl analogue of GW9662, SB1404 (Figure S2) [101], which given its marginal occupation of the *Ω* pocket may prove interesting in the context of screening campaigns similar to that of Ohtera et al. described above [246]. In the design of kinase inhibitors [247, 248], Serafimova et al. and Miller et al. took advantage of the reversibility of the 1,4-addition of thiolates to highly activated electrophiles, such as acrylonitriles [249]. Using this strategy, Kim et al. recently prepared and tuned a series of reversibly covalent and selective inhibitors of PPAR*γ* Ser273 phosphorylation [250]. In a recent report by Jang et al., the crystal structure of PPAR*γ* in complex with the most promising of the resulting ligands, SB1495 (Figure S2), demonstrated that SB1495 bound primarily to *Ω* pocket, where it stabilized the *β*-sheet region, H2′, and the *Ω* loop [251]. Given its covalent, reversible mode of action, ligands such as SB1495 may thus pave the way for more clinically efficacious, yet nontoxic inhibitors of PPAR*γ* Ser273 phosphorylation.

### 8.2. PPAR*α* and PPAR*β/δ*

GW9662, which targets Cys313(285) in PPAR*γ*, has been reported to also covalently modify PPAR*α* and PPAR*β/δ* [71]. In PPAR*α*, Cys275 appears to be more reactive than its neighbour Cys276 [76], which corresponds to Cys313(285) in PPAR*γ*. The side chain of Cys275 also points more directly into the *Ω* pocket than that of Cys276. Regardless of their position, both these cysteine residues may be favourably utilized in the design of electrophilic ligands for the PPAR*α Ω* pocket.

In the field of PPAR*β/δ*, on the other hand, several members of the 5-trifluoromethyl-2-sulfonylpyridine class of covalent antagonists, such as GSK3787 [72], CC618 [73], and Compound 37 [74] (Figure S4) have been reported. While these ligands differed in their selectivity for PPAR*β/δ* versus the other PPAR subtypes and in their rate of reaction with Cys249 [74], treatment of PPAR*β/δ* with either of these ligands resulted in the formation of a Cys249 5-trifluoromethyl-2-pyridyl thioether [75]. Although the aryl moiety appended to PPAR*β/δ* Cys249 is less bulky than the moieties appended to PPAR*γ* Cys313 by treatment with GW9662, T0070907, or SB1404, this modification still inhibited activation of PPAR*β/δ* by classical agonists whose head groups bind to the AF-2 pocket, such as GW501516 or GW0742 (Figure S4) [72–74, 252]. Treatment of PPAR*β/δ* with CC618 or GSK3787 alone did not induce transcription in reporter gene assays [72, 252] nor did treatment with GSK3787 cause the recruitment of the coactivator MED1 (TRAP220) in a TR-FRET-based assay [252]. In a similar assay, treatment with GSK3787 did however result in a moderate, but statistically significant dissociation from the corepressors NCoR and SMRT compared to apo-PPAR*β/δ* [252]. Whether PPAR*β/δ* covalently modified at Cys249 is still capable of binding additional ligands in its *Ω* pocket has yet to be investigated. More importantly, the transcriptional effects of this type of multiple ligation are also unknown. Thus, for the identification of ligands that bind to the PPAR*β/δ Ω* pocket, other screening methodologies than that of Ohtera et al. [246] described in the previous section may require consideration.

To that end, recent studies of PPAR*γ* have highlighted that ^19^F NMR represents a powerful technique to study the binding of fluorine-containing ligands to both the AF-2 pocket and the *Ω* pocket [68, 107]. Chrisman et al. also demonstrated that the effects of the binding of several classes of nonfluorinated ligands on the conformational dynamics of PPAR*γ* could be characterized with ^19^F NMR, after covalent modification of mutagenetically introduced cysteine residues in H3 and H12 with a trifluoroacetone probe [87]. Thus, in the context of screening for ligands that bind to the *Ω* pocket of PPAR*β/δ* treated with the 5-trifluoromethyl-2-sulfonylpyridine class of covalently modifying ligands, the relative proximity of the Cys249 5-trifluoromethyl-2-pyridylthioether to a ligand binding in the *Ω* pocket suggests that a ^19^F NMR-based assay may be viable.

## 9. Conclusions

Herein, we have described discoveries and collective efforts from recent years that have promoted a more nuanced understanding of the pharmacology of PPAR*γ*—an understanding that has already had important ramifications for both PPAR*γ*-targeting therapy and further drug development. These achievements have occurred within a framework that integrates the study of ligand binding, PTMs, protein-protein interactions, and their combined effects on transcription. Collectively, the results obtained within this framework have signalled a shift in interest, away from classical PPAR*γ* agonists that induce supraphysiological levels of unselective transcriptional activation, to the development of nonagonistic- and other noncanonical ligands that more selectively influence gene expression patterns by modulating the occurrence of PTMs. A common theme among many of these new ligands is their binding to the PPAR*γ Ω* pocket. We have ventured to show that the application of these emerging principles to the study of PPAR*α* and PPAR*β/δ* can potentially provide new insights, e.g., through the identification of ligand-sensitive PTMs and the study of their effects on gene expression patterns, but also through the application of known ligands with alternative binding modes in new assay contexts. The data generated by such efforts would lay the foundation for the development of new generations of drugs targeting PPAR*α* and PPAR*β/δ*.

## Supplementary Materials

Supplementary Materials The chemical structures of the ligands described in the contexts of PPAR*γ*, PPAR*α*, and PPAR*β/δ* are depicted in their order of appearance in Figures S1–S4 in the Supplementary Material.
