# Supplementary material for: The PPAR Ω Pocket: Renewed Opportunities for Drug Development
Source: PPAR Res. 2020 Jul 1;2020:9657380. doi: 10.1155/2020/9657380 (PMC7351019; doi:10.1155/2020/9657380)
Supplement: Supplementary Materials — The chemical structures of the ligands described in the contexts of PPARγ, PPARα, and PPARβ/δ are depicted in their order of appearance in Figures S1–S4 in the Supplementary Material. [file 9657380.f1.pdf]

Supplementary Material

THE PPAR Ω POCKET: RENEWED OPPORTUNITIES FOR DRUG DEVELOPMENT

Åsmund Kaupang,[a*] and Trond Vidar Hansen[a]

[a]Section for Pharmaceutical Chemistry, Department of Pharmacy, University of Oslo, 0316 Oslo, Norway.

[*]Corresponding author; Tel.: +47 22 85 65 09; E-mail: asmund.kaupang@farmasi.uio.no, asmundkaupang@gmx.com

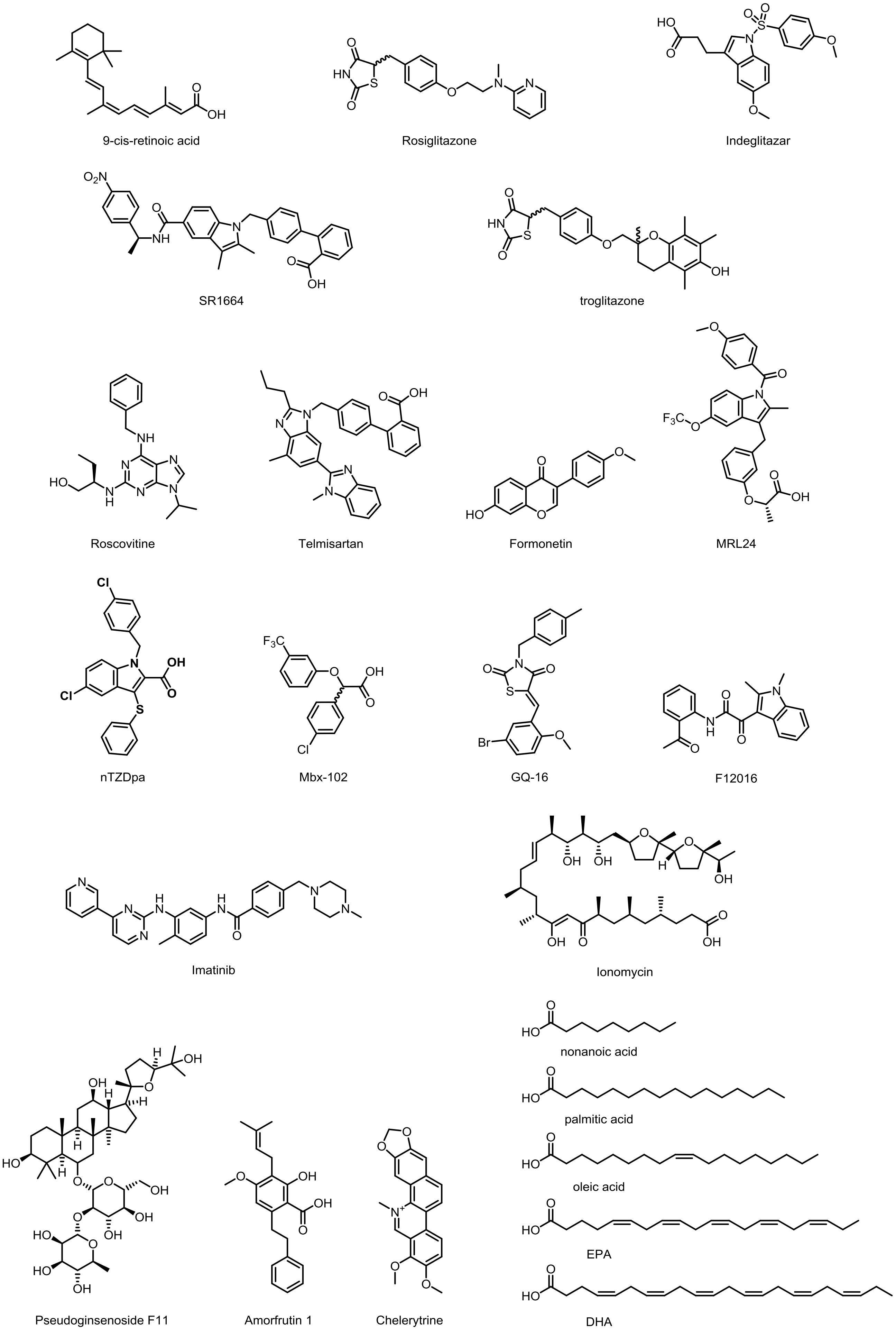

Figure S1. Chemical structures of PPARγ ligands and the Cdk inhibitor roscovitine.

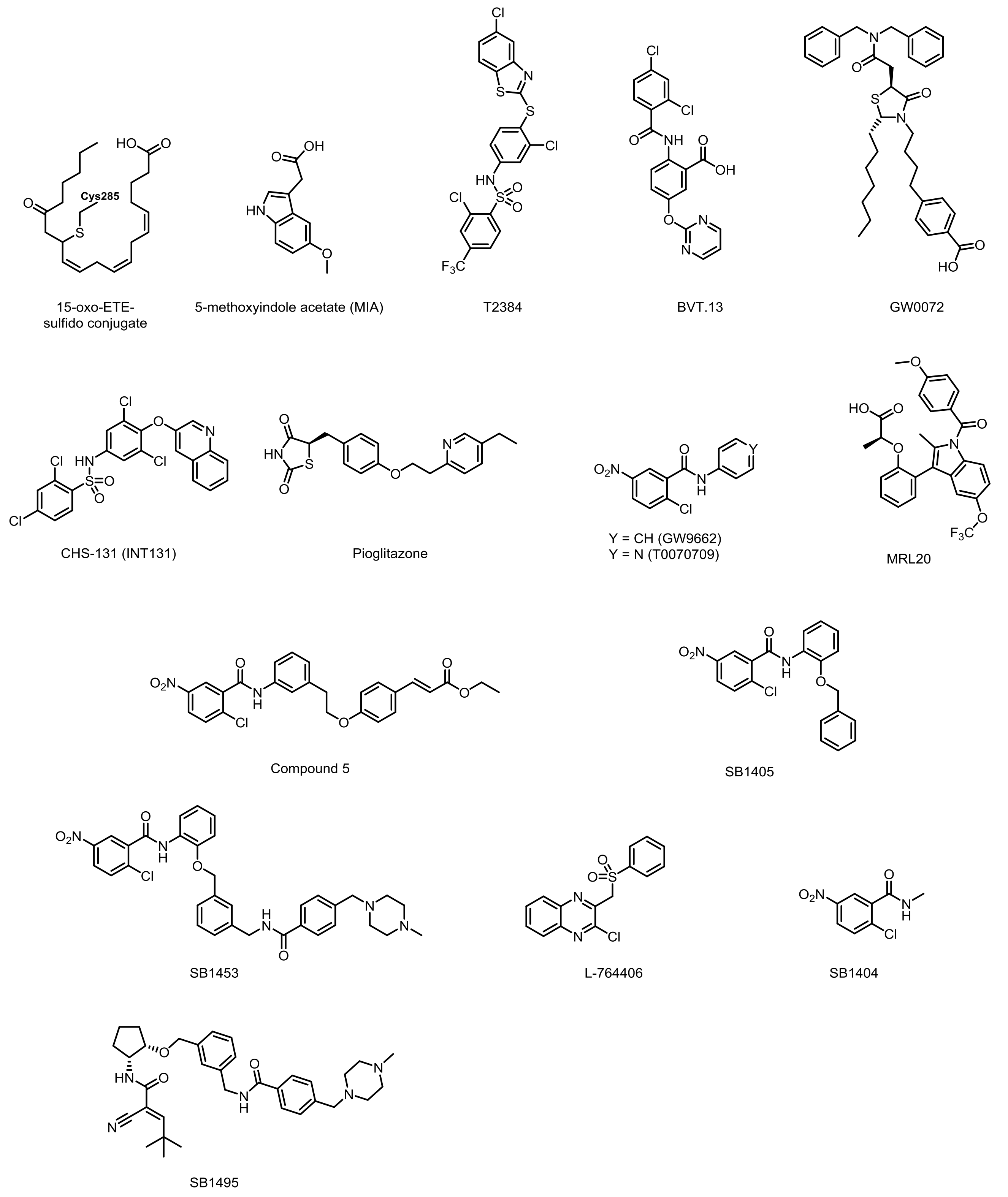

Figure S2. Chemical structures of PPARγ ligands.

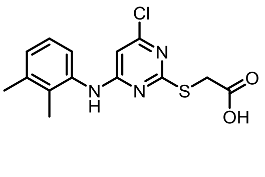

WY14643 (pirinixic acid)

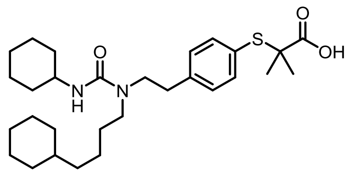

GW7647

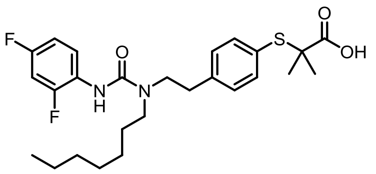

GW9578

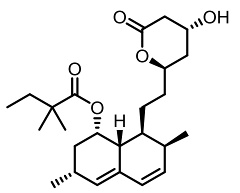

Simvastatin
(δ-lactone form)

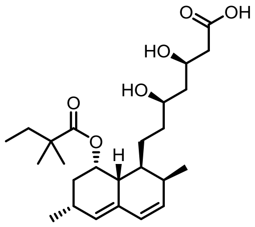

Simvastatin
(β,δ–dihydroxy acid form)

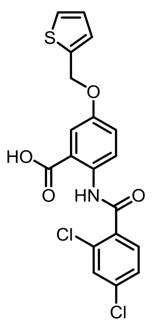

BVT.762 (Cpd. 5a)

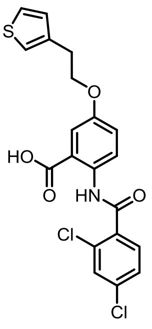

BVT.763 (Cpd. 5b)

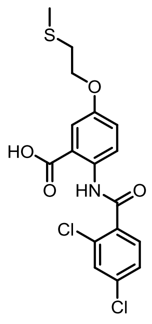

Compound 5d

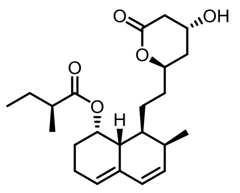

Mevastatin

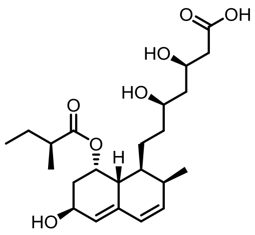

Pravastatin

Figure S3. Chemical structures of PPARα ligands.

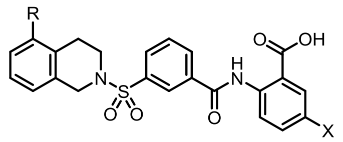

X = R = H (GW9371)
X = Cl, R = COOH (Compound 13)
X = Cl, R = OCH_2COOH (Compound 14)

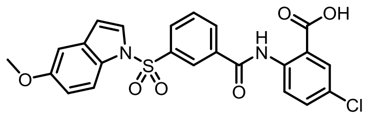

Compound 34

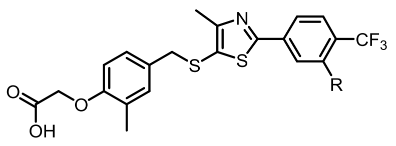

R = H (GW501516)
R = F (GW0742)

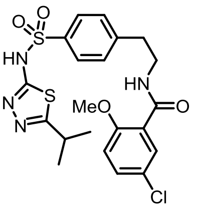

Compound 6

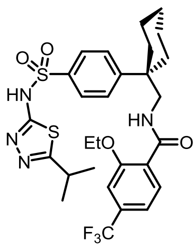

Compound 11j

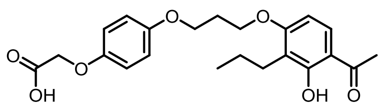

L-165,041

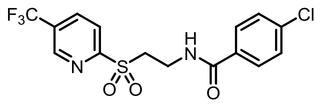

GSK3787

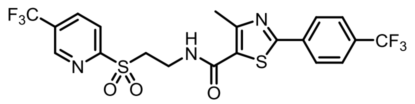

CC618

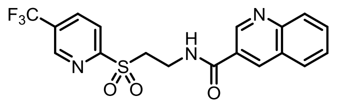

Compound 37

Figure S4. Chemical structures of PPARβ/δ ligands.
